# Post Radical Prostatectomy Erectile Dysfunction. A Single Centre Experience

**DOI:** 10.7759/cureus.34601

**Published:** 2023-02-03

**Authors:** Konstantinos Pikramenos, Maria Zachou, Dimitrios Papadopoulos, Athanasios Papatsoris, Ioannis Varkarakis, Iraklis Mitsogiannis

**Affiliations:** 1 Second Department of Urology, National and Kapodistrian University of Athens, Sismanoglio General Hospital, Athens, GRC; 2 Gastroenterology, National and Kapodistrian University of Athens, Athens, GRC; 3 Gastroenterology, Sismanoglio General Hospital, Athens, GRC; 4 Anaesthesiology, Evgenidio Hospital, National and Kapodistrian University of Athens, Athens, GRC

**Keywords:** urologic oncology, complications, radical prostatectomy, prostate cancer, erectile dysfunction

## Abstract

Introduction: This study aims to determine the effects of radical retropubic prostatectomy on post-operative erectile function.

Materials & Methods: A total of 50 patients were included in this study, diagnosed with localized prostate cancer, and underwent nerve-sparing radical retropubic prostatectomy. All patients completed the International Index of Erectile Function (IIEF-5) questionnaire pre-operatively and on the third, sixth, and twelfth post-operative month and completed a self-reporting of their satisfaction with their sexual performance. Patients with a history of severe heart disease, were on erectile dysfunction medication, or had a score of 7 or less on the IIEF-5 questionnaire, were excluded from the study.

Results: Pre-operatively it was observed that the lower the IIEF-5 score, the higher the biopsy Gleason score. Post-operatively, 16 patients stated that erectile function had returned to the pre-operative IIEF-5 category. In contrast, only 13 of them stated they were happy with their sexual performance on the self-reporting scale. The rest reported dissatisfaction despite returning to their pre-operative erectile function status. IIEF-5 scores were also different when compared amongst the four age groups, with scores indicating that younger age is related to higher IIEF-5 scores. At the 3-month follow-up, no statistically significant difference was observed between age groups. Finally, patients younger than 64 reported significantly less deterioration in post-operative erectile function.

Conclusion: Post-radical prostatectomy erectile dysfunction remains one of the most pressing issues in prostate cancer therapy. A higher Gleason score has a more significant impact on pre-operative ED, and at the same time, the best post-operative ED results are observed in younger patients. Finally, patients need extensive follow-up, therapy, and pre-and post-operative psychological support to have the best possible erectile function.

## Introduction

Prostate cancer (PCa) remains the second cancer-related cause of death in men worldwide [1]. Treatment of organ-confined disease aims to eradicate it completely, with as few complications as possible [2]. Therapy includes Radical Prostatectomy (RP), open, laparoscopic, or robotic, and Radiotherapy [2]. With millions of surgeries done worldwide, RP carries a high risk of post-operative complications, especially Erectile Dysfunction (ED) [3]. Post-operative ED may significantly compromise a patient's quality of life after the surgery and can severely limit the patient's overall satisfaction with therapy [3]. Thus, preserving erectile function after prostate cancer therapy is a hot issue for surgeons and patients alike. In this study, we present our data on ED following RP operations in our department, where more than 150 operations are performed annually.

## Materials and methods

This prospective study was conducted from August 2020 to July 2022 at the 2nd Urology Department of the University of Athens in Sismanoglio General Hospital. The Hospital's Institutional Review Board approved the study, and all patients included signed informed consent.

Of all patients diagnosed with localized PCa and underwent RP in our department, 60 presented with focal, well-differentiated tumors, eligible to undergo a nerve-sparing retropubic technique. They had no history of severe heart disease and were not on ED medication. A detailed sexual history was obtained from all participants before the operation, total Prostate Specific Antigen (PSA) levels were measured for each patient, and all patients were asked to fill out the International Index of Erectile Function (IIEF-5) [4]. Patients were divided into categories based on pre-operation IIEF-5 scores; No ED score of 22-25, mild ED score of 17-21, mild to moderate ED score of 12-16, moderate ED score of 8-11, and severe ED score of 7 or less. Patients scoring 7 or less in the pre-operative evaluation and unable to complete a nerve-sparing procedure intra-operatively were excluded from our final analysis. In total, 50 participants were analyzed. Finally, all participants were asked on a self-reporting scale of "YES" or "NO" to state if they were satisfied with their pre-operative sexual performance, and their biopsy Gleason Score (GS) was noted. GS is a scoring system for prostate cancer, describing cancer cell differentiation and tumor aggressiveness. Each biopsy sample receives two grades, according to its cell differentiation, with the most common and second most common cell pattern placed first and second, respectively. Pathological grades start at 3 for moderately differentiated neoplasms and receive the maximum grade of 5 for poorly differentiated aggressive neoplasms. Possible results are 6=(3+3), 7=(3+4), 7=(4+3), 8=(4+4), 9=(4+5), 9=(5+4), 10=(5+5) [5].

Patients were followed at three, six, and twelve months post-operatively and were asked to state their erectile function and sexual performance according to the scale mentioned above; the IIEF-5 questionnaire was also completed at the same time. Participants have not been prescribed ED medication at any time post-operatively.

Statistical analysis was carried out using the statistical program SPSS 24.0. Mean values, standard deviations, median values, interquartile range (IQR), and histograms were used to describe quantitative variables, whether the data followed the normal distribution or not. The Kolmogorov-Smirnov test was run to check the normality of the distributions. The change in IIEF-5 scores was calculated as the difference of the 12-month IIEF-5 score minus baseline IIEF-5 score and IIEF-5 change among age subgroups was analyzed. Age groups were set as 54-59, 60-64, 65-69, and 70-75 years of age. Non-parametric Kruskal Wallis test was used to determine whether or not there was a statistically significant difference in the baseline IIEF-5 score median among GS subgroups. The same test was run to determine whether or not there was a statistically significant difference in IIEF-5 score medians (at all time points) among age groups. Significance levels were bilateral, and the statistical significance was p<0.05.

## Results

**Figure 1:**
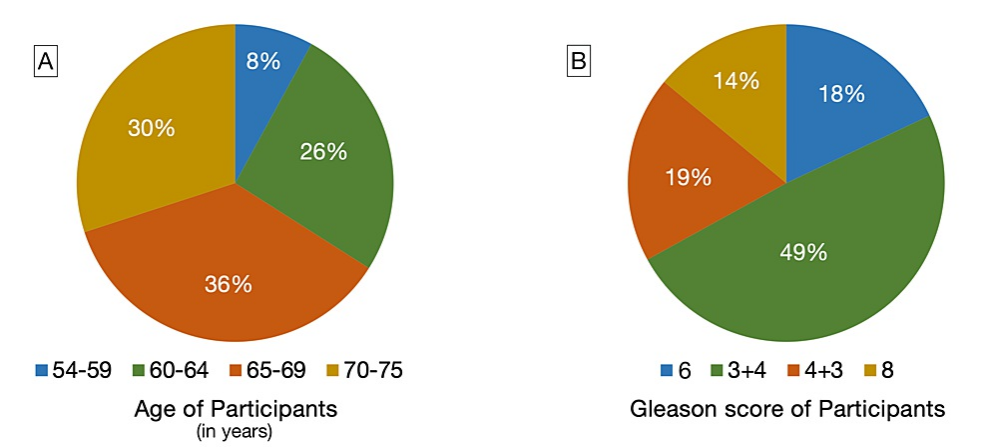
Number of patients in each age group and Gleason score Chart A: Age of participants measured in years (Blue: 54-59 years, Green: 60-64 years, Orange: 65-69 years, Yellow: 70-75 years). Chart B: Gleason score of participants (Blue: Gleason 6, Green: Gleason 3+4=7, Orange: Gleason 4+3=7, Yellow: Gleason 8).

In total, 50 patients with PCa, aged 54-75 (mean 66.3 ± 0.75 years), were included. Participants' age groups and GS group distributions are presented in Figure 1.

**Figure 2:**
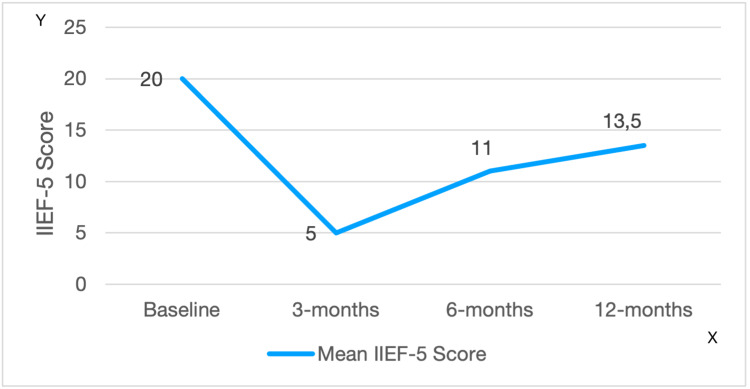
Median IIEF-5 score as it was measured at Baseline, 3rd, 6th and 12th month post-operatively IIEF-5: International Index of Erectile Function Y axis: Mean IIEF-5 Score X axis: Measurement at Baseline and 3rd, 6th, and 12th post-operative month

Pre-operatively, 23 participants (46%) reported no ED (score 22-25). Of those who reported a degree of erectile dysfunction, 12 (24%) had mild ED (score 17-21), 9 (18%) had mild to moderate ED (score 12-16), and 6 had moderate ED (score 8-11); no patient had severe ED (score ≤7) pre-operatively. On the self-reporting scale, 32 participants (64%) were satisfied with their performance. Results of ED, before and at specific time points after the operation are presented in Table 1. Median IIEF-5 scores are presented in Figure 2.

**Table 1:** Percentage of patients with erectile dysfunction as measured at Baseline, 3rd, 6th, and 12th months post-operatively ED: Erectile Dysfunction

ED	Baseline	3 months post-operatively	6 months post-operatively	12 months post-operatively
No	46%	0%	4%	16%
Mild	24%	0%	14%	16%
Mild to moderate	18%	2%	24%	36%
Moderate	12%	24%	30%	16%
Severe	0%	74%	28%	16%

**Figure 3:**
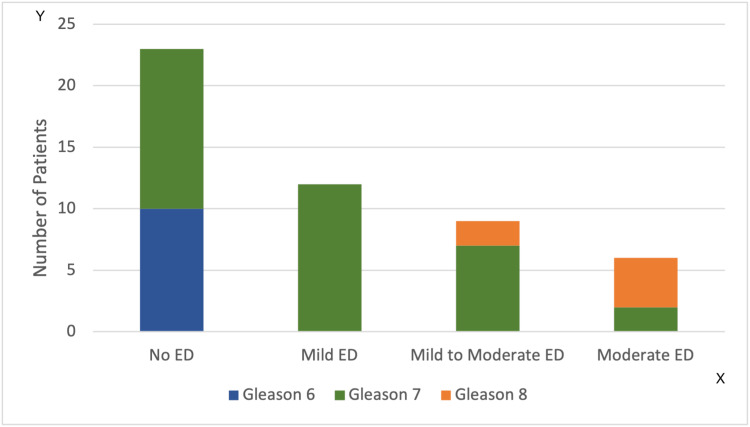
Biopsy Gleason Score Compared to Erectile Dysfunction ED: Erectile Dysfunction Y-axis: Number of patients X-axis: ED categories (No, Mild, Mild to Moderate, Moderate) Blue: Gleason 6, Green: Gleason 7, Orange: Gleason 8

There was a statistically significant difference in pre-operative IIEF-5 scores amongst GS subgroups (p=0.002); the higher the biopsy GS score, the lower the pre-operative IIEF-5 score was observed (Figure 3). Post-operatively there was no statistical difference between GS score subgroups. 

Post-operatively, 19 patients (38%) stated they were satisfied with their sexual performance by answering YES on the self-reporting questionnaire. In contrast, in 16 (32%), erectile function had returned to the pre-operative (baseline) IIEF-5 category. Of these 16 patients, 13 (81%) stated they were happy with their sexual performance, whereas the rest, 3 (19%), reported dissatisfaction with this issue, despite having returned to their pre-operative erectile function status. 

**Figure 4:**
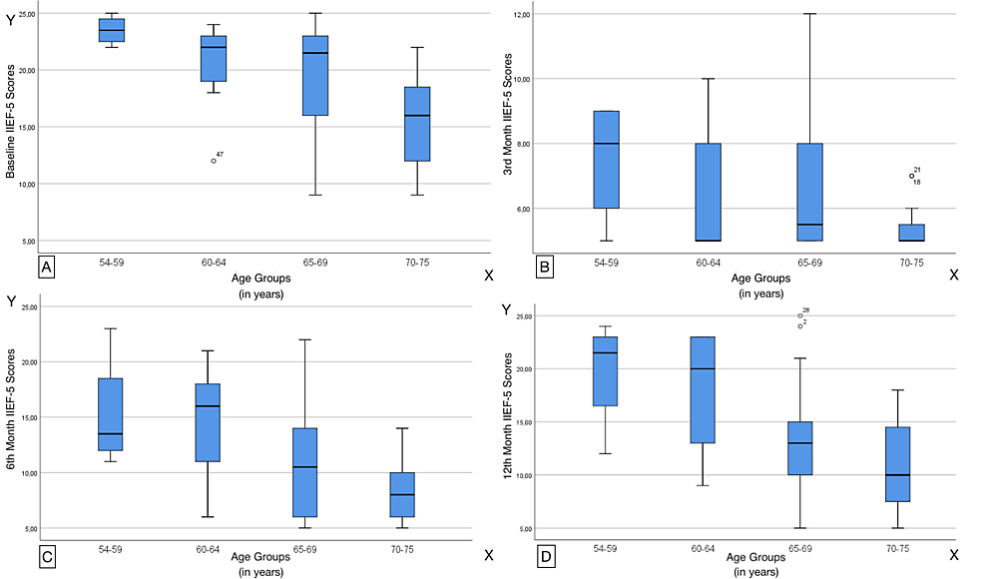
IIEF-5 scores in all age groups at Baseline and 3rd, 6th, and 12th post-operative months. (Kruskal Wallis test) IIEF-5: International Index of Erectile Function Y-axis: IIEF-5 Scores X-axis: Age Groups measured in years Diagram A: Measurement at Baseline Diagram B: Measurement at 3 months post-operative, Diagram C: Measurement at 6 months post-operative, Diagram D: Measurement at 12 months post-operative.

IIEF-5 scores differed among the four age groups (Figure 4). Baseline (p=0.002), 6-month (p=0.005), and 12-month (p=0.012) scores indicated younger age to be related to higher IIEF-5 scores. However, at the 3-month follow-up, no statistically significant difference was observed, as all age groups reported statistically similar IIEF-5 scores (p= 0.119).

Table 2 shows the change in IIEF-5 score in each patient age group at the 12-month follow-up; age groups 54-59 and 60-64 reported a significantly smaller deterioration in post-operative erectile function (p=0.07).

**Table 2:** IIEF-5 change at 12 months for each age group IIEF-5: International Index of Erectile Function Age measured in years IIEF-5 change = 12 month IIEF-5 score - Baseline IIEF-5 Score

Age (in years)	IIEF-5 Change (12 months - Baseline)
54-59	-1 (8.25)
60-64	-1 (8.5)
65-69	-5.5 (7.5)
70-75	-4 (5)

## Discussion

Several studies have evaluated erectile function after RP reporting contradictory results. Many factors may influence erectile function status post-RP, including biopsy GS and center experience [6,7]. We have found age to be a significant factor for recovery of erectile function after RP, as younger patients (p=0.07) reported significantly smaller changes in post-operative IIEF scores after the sixth post-operative month. Given that, at the three-month follow-up, erectile function was similarly decreased across all age groups, one might expect it to improve after the sixth post-operative month at the earliest. 

Bratu O et al. [8] reported in their review an estimated sexual function recovery around 12 to 24 months after surgery, with the help of any non-specific ED therapy. Moskovic et al. [9] reviewed the current literature for all possible therapies for post-op ED. Ultimately, they described the "Baylor College of Medicine Erectile Preservation Program" [9], a combination of phosphodiesterase-5 inhibitors, intraurethral alprostadil suppositories, intra-cavernosal injections, hormone replacement therapy, and vacuum erection devices that can speed up erectile recovery after radical prostatectomy. In our study, no form of treatment was given to patients pre- or post-operatively; thus, any restoration of erectile function was attributed to being a result of the surgical technique alone.

We have also demonstrated an inverse correlation between GS and pre-operative IIEF-5 scores, as we have found that the higher the biopsy GS, the lower the pre-operative IIEF-5 score. This may imply that PCa is a potential cause of ED. Similar findings have been reported by others as well [7]. In a study by Jeong et al. [7], it was reported that patients with severe pre-operative ED (IIEF-5 <8) had larger tumors and higher GS in the final pathology. The authors hypothesized that this finding could be attributed to altered hormonal levels (testosterone or sex hormone-binding globulin) [7]. Low testosterone level, which adversely affects erectile function, has been correlated with higher tumor volume and worse prognosis [10] and an extra-prostatic disease [11]. Therefore, decreased erectile function can be used as a predictor for PCa aggressiveness. However, this observation can also be attributed to the higher probability of lower IIEF-5 scores [12] and higher GS as males grow older [13]. As we cannot fully understand this observation, further research could provide a more definite conclusion.

In our study, only 46% of participants had normal erectile function pre-operatively, and only 64% stated they were satisfied with their performance. ED is a common disorder over 40, with an exponential increase every decade, and may co-exist with prostate cancer [12,14,15]. Hence, assessment of patients' erectile function pre-operatively is crucial and allows physicians, based on this, to provide a proper consultation on what a patient should realistically expect following the operation. 

Interestingly, of all the eligible participants, 16 returned to their baseline IIEF-5 after 12 months. However, only 81% of them were happy with their sexual performance. This discrepancy indicates that other factors (i.e., psychological) may influence patients perceived sexual performance. Even after the return of a patient's IIEF-5 score to the pre-operative levels, he may not feel as before. Hence, psychological support may be necessary for patients, as they might have perception issues post-surgery as the cause of their ED. It is well documented that erectile function can be of organic or psychological origin [16-18] and may be intensified by major surgery.

Finally, this study has its limitations. The small number of participants did not allow a deeper exploration of the possible correlation between GS and IIEF-5 scores, a psychological evaluation, pre-and post-operatively, would permit a better assessment of the influence of a patient's psychological status on their erectile function and the inclusion of a separate subgroup with patients receiving a penile rehabilitation protocol, would clarify more clearly its effects on post-operative erectile function.

## Conclusions

Post-radical prostatectomy erectile dysfunction remains one of the most pressing issues in prostate cancer therapy, and patients are deeply concerned about their post-operative sexual performance. The effects of surgery on erectile function are significant, with only 32% of patients returning to their pre-operative function. Younger patients seem to have quicker recovery and better overall results, yet an erectile rehabilitation program for all patients might be necessary. Furthermore, erectile dysfunction seems to be a widespread pre-existing problem in the community. At the same time, higher cancer Gleason Scores seem to have a possible effect on pre-operative erectile function as well. Thus, it is vital for careful patient consultations, determination of any pre-existing erectile dysfunction, and detailed discussions about the operation and its complications with the patients for a more holistic approach that will provide patients with realistic expectations, psychological support, and better overall post-treatment satisfaction.
